# New *Lycopodium* alkaloids from *Lycopodium obscurum*

**DOI:** 10.1007/s13659-013-0015-x

**Published:** 2013-04-01

**Authors:** Xue-Yuan Zhang, Liao-Bin Dong, Fei Liu, Xing-De Wu, Juan He, Li-Yan Peng, Huai-Rong Luo, Qin-Shi Zhao

**Affiliations:** 115 State Key Laboratory of Phytochemistry and Plant Resources in West China, Kunming Institute of Botany, Chinese Academy of Sciences, Kunming, 650201 China; 215 University of Chinese Academy of Sciences, Beijing, 100049 China

**Keywords:** obscurumines, *Lydopodium* alkaloids, *Lycopodium obscurum*, acetylcholinesterase (AChE) inhibitory activity

## Abstract

Three new *Lycopodium* alkaloids, obscurumines C-E (**1–3**), along with nine known compounds, were isolated from the club moss *Lycopodium obscurum* L. Structures of the new compounds were determined on the basis of their spectroscopic analysis and the relative configurations of **1** were established by X-ray crystallographic analysis. All the new isolates were tested for the acetylcholinesterase (AChE) inhibitory activity.

**Electronic Supplementary Material:**

Supplementary material is available for this article at 10.1007/s13659-013-0015-x and is accessible for authorized users.

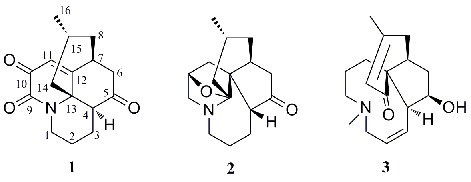

## Introduction

The *Lycopodium* alkaloids represent a large family of plant secondary metabolites obtained from the club moss belonging to Lycopodiaceae.[1,2] Since the impressively diverse skeletons and interesting biological activities, these alkaloids continue to be of interest from a biogenetic and biological point of view and some of them have been challenging targets for total synthesis.[3–5] *Lycopodium obscurum* L., one of the club moss, has been used in China as a traditional folk medicine for the treatment of contusion, dysmenorrhea, quadriplegia, and arthritic pain.[6,7] Previously phytochemical investigation indicated triterpenoids were the main compounds of this plant and only few *Lycopodium* alkaloids were isolated.[8–10] As part of an ongoing program aimed at discovering structurally interesting and bioactive *Lycopodium* alkaloids,[11,12] three new *Lycopodium* alkaloids, obscurumines C–E (**1**–**3**), along with nine known compounds, were isolated from the whole herb of *L. obscurum* L. Herein, we report the isolation, structure elucidation, and acetylcholinesterase (AChE) inhibitory activity of the new isolates.

## Results and Discussion

The alkaloidal extract of *L. obscurum* was separated by normal-phase silica gel, RP-18 silica gel, HPLC, and Sephadex LH-20 chromatography to afford three new *Lycopodium* alkaloids, obscurumines C–E (**1**–**3**), along with nine known compounds. The structures of known compounds, compared with literatures data, were identified as lycopodine,[13] anhydrolycodoline,[14] obscurinine,[9] obscurinine B,[8] lycoflexine,[15] acetyldihydrolycopodine,[16] (+)-acetylfawcettiine,[17] *N*-demethyl-*α*-obscurine,[18] *N*-demethyl-*β*-obscurine.[19]

**Figure 1.** Chemical structure of isolated compounds **1**–**3**

Obscurumine C (**1**), colorless crystals, has a molecular formula $C_{16}H_{19}NO_3$ as established by HREIMS at m/z 273.1359 [$M^+$] (calc. 273.1365), indicating eight degrees of unsaturation. The IR absorptions at 1710, 1685, and 1658 $cm^{-1}$ implied the presence of carbonyl groups. Analysis of the $^1H$ and $^{13}C$ NMR spectra of **1** (Table 1) revealed 16 carbon signals due to five quaternary carbons (three ketone carbons, one olefinic carbon, and one $sp^3$ quaternary carbon), four methine groups (one olefinic carbon), six methylene groups, and one methyl group ($\delta_H$ 0.71, d, $J$ = 5.9 Hz). The $^1H$-$^1H$ COSY correlations revealed the existence of two fragments, **a** (C-1–C-4) and **b** (C-6–C-8, C-8/C15/C-14, C-15–C-16), as shown in Figure 2. In the HMBC spectrum, the correlations of the proton ($\delta_H$ 2.42, H-4) at the terminal carbon (C-4) of fragment **a** and the protons ($\delta_H$ 2.71, 2.39, H-6) at the terminal carbon (C-6) of fragment **b** with the carbonyl carbon (C-5, $\delta_C$ 205.7) indicated the connection of C-4/C-5/C-6. While, the connection of C-4/C-13/C-14 were derived from HMBC cross-peaks of H-4 and H-14 to C-13. Meanwhile, the HMBC correlations of H-1 with C-9 and C-13 established the connections of C-1,

*To whom correspondence should be addressed. E-mail: qinshizhao@mail.kib.ac.cn

**Table 1.** $^1$H and $^{13}$C NMR data for **1**–**3** ($\delta$ in ppm and $J$ in Hz)

| No. | 1[a] | | 2[b] | | 3[c] | |
|---|---|---|---|---|---|---|
| | $\delta_C$ | $\delta_H$ | $\delta_C$ | $\delta_H$ | $\delta_C$ | $\delta_H$ |
| 1a | 36.6 $CH_2$ | 4.51 (dd, 13.8, 3.9) | 41.9 $CH_2$ | 3.05 (m) | 52.4 $CH_2$ | 3.32 (dd, 13.5, 7.9) |
| 1b | | 2.79 (dt, 13.8, 3.1) | | 2.86 (m) | | 2.92 (dd, 13.5, 5.6) |
| 2a | 24.4 $CH_2$ | 1.73 (br. d, 16.3) | 20.2 $CH_2$ | 1.98 (m) | 129.4 CH | 5.72 (overlap) |
| 2b | | 1.31 (m) | | 1.47 (m) | | |
| 3a | 19.0 $CH_2$ | 1.99 (d, 16.3) | 19.4 $CH_2$ | 2.16 (m) | 131.8 CH | 5.64 (t, 10.8) |
| 3b | | 1.51 (overlap) | | 1.37 (m) | | |
| 4 | 57.1 CH | 2.42 (dd, 10.9, 2.6) | 46.4 CH | 2.32 (dd, 11.8, 2.3) | 47.1 CH | 3.95 (dd, 10.8, 6.5) |
| 5 | 205.7 C | | 218.2 C | | 70.5 CH | 3.83 (br. d, 6.1) |
| 6a | 46.0 $CH_2$ | 2.71 (dd, 20.5, 8.2) | 42.0 $CH_2$ | 2.28 (dd, 18.9, 8.0) | 37.6 $CH_2$ | 1.79 (m) |
| 6b | | 2.39 (d, 20.5) | | 1.93 (d, 18.9) | | 1.64 (dt, 13.8, 9.7) |
| 7 | 40.2 CH | 3.04 (br. s) | 38.2 CH | 2.17 (m) | 40.4 CH | 2.34 (m) |
| 8a | 43.2 $CH_2$ | 1.80 (d,15.0) | 34.3 $CH_2$ | 1.39 (m) | 30.8 $CH_2$ | 2.49 (overlap) |
| 8b | | 1.34 (m) | | 1.39 (m) | | 2.10 (d, 19.4) |
| 9a | 155.8 C | | 50.2 $CH_2$ | 3.02 (m) | 56.7 $CH_2$ | 2.67 (d, 9.4) |
| 9b | | | | 2.58 (d, 8.2) | | 2.49 (overlap) |
| 10a | 177.0 C | | 76.5 CH | 4.68 (t, 4.7) | 20.9 $CH_2$ | 1.21 (dt, 11.0, 7.1) |
| 10b | | | | | | 1.21 (dt, 11.0, 7.1) |
| 11a | 121.5 CH | 6.25 (s) | 39.5 $CH_2$ | 1.73 (d, 12.2) | 31.4 $CH_2$ | 1.71 (m) |
| 11b | | | | 1.50 (m) | | 1.31 (m) |
| 12 | 167.3 C | | 57.9 C | | 57.4 C | |
| 13 | 63.1 C | | 96.5 C | | 201.4 C | |
| 14a | 44.0 $CH_2$ | 2.64 (d,16.3) | 29.9 $CH_2$ | 2.07 (dd, 15.0, 5.9) | 124.1 CH | 5.72 (overlap) |
| 14b | | 1.02 (t, 16.3) | | 1.69 (br. d, 15.0) | | |
| 15 | 24.5 CH | 1.51 (overlap) | 27.4 CH | 2.21 (m) | 158.9 C | |
| 16 | 20.9 $CH_3$ | 0.71 (d, 5.9) | 19.2 $CH_3$ | 1.20 (d, 7.4) | 24.1 $CH_3$ | 1.87 (s) |
| *N*-$CH_3$ | | | | | 43.1 $CH_3$ | 2.29 (s) |

[a]Recorded in $CDCl_3$ at 500 MHz for $^1$H and 125 MHz for $^{13}$C; [b]Recorded in $CDCl_3$ at 600 MHz for $^1$H and 150 MHz for $^{13}$C; [c]Recorded in DMSO at 600 MHz for $^1$H and 150 MHz for $^{13}$C.

C-9, and C-13 through a nitrogen atom. The above analysis indicated **1** should be a lycopodine-type alkaloid with many similarities to that of anhydrolycodoline,[14] a known compound also isolated during the isolation. The obvious differences were that **1** possessed two more carbonyl groups one of which was connected to C-9 ($\delta_C$ 155.8) as deduced from the HMBC correlations of H-1 and H-11 with C-9. The other carbonyl group was speculated to attached at C-10 according to the $^{13}$C chemical shift and confirmed by the HMBC correlations of H-11 to C-10. Thus, the gross structure of obscurumine C (**1**) was elucidated to be **1** possessing a lycopodine-type skeleton with a scarce two adjacent ketones unit.

The relative configuration of **1** was established by ROESY experiment. In the ROESY spectrum, the correlations of H-4 with H-6b and H-6a with H-15 were observed which indicated the $\alpha$-orientation of H-4 and Me-16. Since the two adjacent ketone unit is scarce in *Lycopodium* alkaloid and to confirm the structure and the relative configuration, a X-ray crystallographic analysis of **1** (Figure 2) was carried out which unambiguously certificated the structure and the relative configurations of compound **1**.

Compound **2** was obtained as colorless oil. The HREIMS analysis gave an m/z at 261.1735 ([M$^+$]) (calc. 261.1729) that established the molecular formula $C_{16}H_{23}NO_2$, corresponding to six degrees of unsaturation. The $^{13}$C NMR spectrum displayed 16 carbon signals due to one methyl, eight

**Figure 2.** Key 2D NMR correlations and X-ray structure of compound **1**

methylene, four methine, and three quaternary carbons. Among them, one *sp*$^3$ quaternary carbon ($\delta_C$ 96.5) was ascribed to the carbon (C-13) which bearing both an oxygen atom and a nitrogen atom and one *sp*$^2$ quaternary carbons were attributable to the ketone group ($\delta_C$ 218.2). Partial structures a–c (C-1–C-4, C-6–C-8/C-15, and C-9–C-11) were deduced from $^1$H-$^1$H COSY (Figure 3) of **2**. Further detailed 2D NMR analysis suggested compound **2** was a fawcettimine-type alkaloid which was similar to that of fawcettimine.[20] The obvious difference was that **2** has one oxygened methine group rather than a methylene group in fawcettimine, which was connected to C-10 as deduced from the $^1$H-$^1$H COSY

correlations of $H_2$-9/H-10/$H_2$-11 and confirmed by the HMBC correlations of H-10 with C-9, C-11, and C-12. Additional, the HMBC correlation of H-10 with C-13 as well as the molecular formula of **2** indicated the connection of C-10 and C-13 through an oxygen atom. In ROESY spectrum, the cross peaks of H-2b/H-4 and H-4/H-14a were observed, which suggested H-4 was $\beta$-oriented. H-15 was determined to be axially oriented based on the small coupling constant between H-15 and axial H-14a [dd, 15.0 ($^3J_{H14a\text{-}H14b}$), 5.9 ($^3J_{H14a\text{-}H15}$) Hz)] and the small coupling constant between H-15 and equatorial H-14b [br. d, 15.0 ($^3J_{H14a\text{-}H14b}$) Hz]. Meanwhile, the relative configuration of H-10 was suggested to be $\alpha$ according to the rigidity of compound **2**. Therefore, the structure of **2** was established as shown in Figure 1 and named as obscurumine D.

**Figure 3.** Key 2D NMR correlations of compound **2**

Obscurumine E (**3**) has a molecular formula $C_{17}H_{25}NO_2$ according to the HREIMS [m/z 275.1891 ([M$^+$]), calc. 275.1885] spectrum, implying six degrees of unsaturation. The IR spectrum showed absorption bands for hydroxyl (3439 cm$^{-1}$) and ketone (1640 cm$^{-1}$) groups. The $^{13}$C NMR and DEPT spectra revealed the existence of seventeen carbons due to three quaternary carbons, six methine groups, six methylene groups, and two methyl groups**.** Detailed analysis of its 1D and 2D NMR data suggested that **3** was similar to that of *epi*-lobscurinol.[18] The only difference was that the double bond between C-3/C-4 in *epi*-lobscurinol was shift to C-2/C-3 as deduced from the $^1$H-$^1$H COSY correlations of H-1/H-2/H-3/H-4 and confirmed by the HMBC correlations of H-2 with C-1 and C-4. In the ROESY spectrum, the correlations of H-4/H-11b and H-5/H-7 were observed which suggested the relative configuration of **3** was the same as *epi*-lobscurinol. So, the structure of **3** was deduced and shown in Figure 1.

The new compounds obscurumine C–E (**1**–**3**) were tested for acetylcholine esterase (AChE) inhibitory activity. However, none of them showed obvious activity.

## Experimental Section

**General Experimental Procedures.** Melting points were obtained on an X-4 micro melting point apparatus. Optical rotations were measured on a JASCO-20C digital polarimeter. UV spectra were recorded using a Shimadzu UV-2401A spectrophotometer. IR spectra were obtained on a Tensor 27 spectrometer with KBr pellets. 1D and 2D NMR spectra were performed on Bruker AM-400, DRX-500, or AVANCE III-600 spectrometers with TMS as an internal standard. Mass spectra were taken on VG Auto Spec-3000 or API-Qstar-Pulsar instruments. X-ray diffraction was performed on a Bruker APEX DUO diffractometer using graphitemonochromated Mo K$\alpha$ radiation. Column chromatography (CC) was performed using silica gel (100–200 and 200–300 mesh, Qingdao Marine Chemical Co. Ltd., Qingdao, China), MCI gel (CHP 20P, 75–150 $\mu$m; Mitsubishi Chemical Co., Japan), and Sephadex LH-20 (Amersham Pharmacia Biotech, Sweden).

**Plant Material.** The whole plants of *L. obscurum* were collected from Guizhou Province, China, and identified by Professor Xiao Cheng. A voucher specimen (Lo 2011081501) was deposited with the State Key Laboratory of Phytochemistry and Plant Resources in West China, Kunming Institute of Botany, Chinese Academy of Sciences.

**Extraction and Isolation.** Air-dried and pulverised plants of *L. obscurum* were extracted three times with methanol under reflux at 50 °C and the combined extract was removed under vacuum to afford a viscous residue. The crude extract was adjusted at pH 2 with 10% hydrochloric acid and then partitioned twice with acetic ether. Water-soluble materials, which were adjusted at pH 10 with saturated NaOH, were extracted three times with chloroform. The chloroform-soluble materials (479 g) were chromatographed over silica gel CC (32 × 120 cm) eluting with a gradient mobile phase (petroleum ether-acetone 9:1, 8:2, 7:3, 6:4, then methanol) to give 3 major fractions, Fr.A–Fr.C. Fr.A (29 g) was chromatographed on a reversed-phase preparative MPLC (MCI, 70 × 460 mm) column using step-gradient elution with a mixture of methanol/water (v/v, 70% to 100% methanol) to give three subfractions, Fr.A1–Fr.A3. Fr.A1 (13 g) was separated by repeat silica gel CC (4.6 × 50 cm) and recrystalization to offer **1** (230 mg) as well as anhydrolycodoline (2.1 g). Fr.B (159 g) was subjected to a reversed-phase preparative MPLC (MCI, 70 × 460 mm) column with a mixture solvent of methanol/water (v/v, 40% to 100% methanol) to yield five subfractions. Fr.B3 (41 g) was eluted on silica gel CC (6.0 × 60 cm) with acetic ether-acetone (92:8) to yield a large number of lycopodine (30 g), obscurumine B (5.4 g), (+)-acetylfawcettiine (49 mg) and obscurinine (21 mg). Fr.B4 (1.2 g) was purified by repeat silica gel CC (2.6 × 40 cm) to afford **2** (46 mg). Fr.C (136 g) was subjected to gradient elution on a reversed-phase preparative MPLC (MCI, 70 × 460 mm) column using a mixture solvent of methanol/water (v/v, 10% to 80% methanol) to yield three subfractions, Fr.C1–Fr.C3. Fr.C3 (4.2 g) was eluted with chloroform-methanol (4:1) on silica gel CC (3.0 × 40 cm) to yield N-demethyl-$\beta$-obscurine (890 mg). Fr.C2 (8.6 g) was separated on silica gel CC (4.0 × 40 cm) using petroleum ether-aceticether-diethylamine (10:1:1) to give acetyldihydrolycopodine (173 mg), lycoflexine (286 mg) and another fraction which was eluted with chloroform-methanol (2:3) to yield **3** (36 mg) and *N*-demethyl-$\alpha$-obscurine (938 mg).

**Obscurumine C (1):** colorless crystal; mp 195–196 °C; $[\alpha]^{25.8}_{D}$ – 58.8 (*c* 0.35, MeOH); UV (MeOH): $\lambda_{max}$ (log ε) 250 (3.46), 201 (3.38) nm; IR (KBr): $\nu_{max}$ 2964, 1710, 1685, 1658, 1414, 1353, 1287, 1227 cm$^{-1}$; $^1$H and $^{13}$C NMR data, see Table 1; ESIMS: m/z 274 [M + H]$^+$; HREIMS: m/z 273.1359 [M$^+$] (calc. for $C_{16}H_{19}NO_3$ 273.1365).

**Obscurumine D (2):** colorless oil; $[\alpha]^{25.8}_{D}$ – 126.6 (*c* 0.18, MeOH); UV (MeOH): $\lambda_{max}$ (log ε) 204 (2.48) nm; IR (KBr): $\nu_{max}$ 2932, 1737 (ketone) 1641, 1454, 1126 cm$^{-1}$; $^1$H and $^{13}$C

NMR data, see Table 1; ESIMS: m/z 262 $[M + H]^+$; HREIMS: m/z 261.1735 $[M^+]$ (calc. for $C_{16}H_{23}NO_2$ 261.1729).

**Obscurumine E (3):** colorless oil; $[\alpha]^{25.8}_{D}$ + 100.0 (*c* 0.15, MeOH); UV (MeOH): $\lambda_{max}$ (log ε) 238 (3.47) nm; IR (KBr): $\nu_{max}$ 3439, 1640 $cm^{-1}$; $^1H$ and $^{13}C$ NMR data, see Table 1; ESIMS: m/z 276 $[M + H]^+$; positive ion HREIMS: m/z 275.1891 $[M^+]$ (calc. for $C_{17}H_{25}NO_2$ 275.1885).

**Crystal Data for Obscurumine C (1):** $C_{16}H_{19}NO_3$, $M$ = 273.32; orthorhomic, space group $P2_12_12_1$; $a$ = 8.1260(8) Å, $b$ = 12.8706 (12) Å, $c$ = 12.9226 (12) Å, $\alpha$ = 90.00, $\beta$ = 90.00, $\gamma$ = 90.00, $V$ = 1351.5 (2) $Å^3$, $Z$ = 4, $\mu$ (Mo$K\alpha$) = 0.093 $mm^{-1}$, crystal dimensions 0.87 × 0.36 × 0.30 mm was used for measurement on a Bruker APEX DUO diffractometer using graphitemonochromated Mo K$\alpha$ radiation. The total number of reflections measured was 9380, of which 4335, were observed, $I > 2\sigma(I)$. Final indices: $R_1$ = 0.0599, $wR_2$ = 0.1719. Crystallographic data for the structure of **1** have been deposited in the Cambridge Crystallographic Data Centre (deposition number CCDC 908305). Copies of the data can be obtained free of charge from the CCDC via www.ccdc.cam.ac.uk.

**Acetylcholinesterase Inhibition.** Acetylcholinesterase (AChE) inhibitory activity of the compounds isolated was assayed by the spectrophotometric method developed by Ellman et. al. with slightly modification.[21] *S*-Acetylthiocholine iodide, *S*-butyrylthiocholine iodide,5,5′-dithio-bis-(2-nitro-benzoic) acid (DTNB, Ellman's reagent), acetylcholinesterase derived from human erythrocytes were purchased from Sigma Chemical. Compounds were dissolved in DMSO. The reaction mixture (totally 200 $\mu$L) containing phosphate buffer (pH 8.0), test compound (50 $\mu$M), and acetyl cholinesterase (0.02 U/mL), was incubated for 20 min (30 °C). Then, the reaction was initiated by the addition of 40 $\mu$L of solution containing DTNB (0.625 mM) and acetylthiocholine iodide (0.625 mM) for AChE inhibitory activity assay, respectively. The hydrolysis of acetylthiocholine was monitored at 405 nm every 30 seconds for one hour. Tacrine was used as positive control with final concentration of 0.333 $\mu$M. All the reactions were performed in triplicate. The percentage inhibition was calculated as follows: % inhibition = (E – S)/E × 100 (E is the activity of the enzyme without test compound and S is the activity of enzyme with test compound).

## Electronic Supplementary Material

Supplementary material is available in the online version of this article at http://dx.doi.org/ 10.1007/s13659-013-0015-x and is accessible for authorized users.

## Acknowledgments

This work was financially supported by the National Basic Research Program of China (973 Program Nos. 2011CB915503 and 2009CB522303) and the National Natural Science Foundation of China (Nos. U0932602 and 90813004).

**Open Access** This article is distributed under the terms of the Creative Commons Attribution License which permits any use, distribution, and reproduction in any medium, provided the original author(s) and source are credited.

## Electronic supplementary material

Supplementary material, approximately 1.94 MB.

Supplementary material, approximately 15.8 KB.
