# Supplementary material for: New Lycopodium alkaloids from Lycopodium obscurum
Source: Nat Prod Bioprospect. 2013 Apr 1;3(2):52–5. doi: 10.1007/s13659-013-0015-x (PMC4131663; doi:10.1007/s13659-013-0015-x)
Supplement: Supplementary file 1 — Supplementary material, approximately 1.94 MB. [file 13659_2013_15_MOESM1_ESM.pdf]

Electronic Supplementary Material

New *Lycopodium* alkaloids from *Lycopodium obscurum*

Xue-Yuan ZHANG,[a,b] Liao-Bin DONG,[a,b] Fei LIU,[a,b] Xing-De WU,[a] Juan HE,[a] Li-Yan PENG,[a] Huai-Rong LUO,[a] and Qin-Shi ZHAO[a,*]

[a]State Key Laboratory of Phytochemistry and Plant Resources in West China, Kunming Institute of Botany, Chinese Academy of Sciences, Kunming 650201, China

[b]University of Chinese Academy of Sciences, Beijing 100049, China

Received 19 February 2013; Accepted 17 March 2013

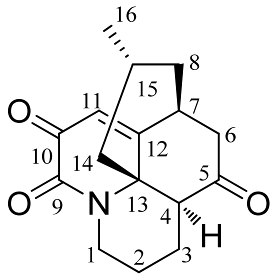

1

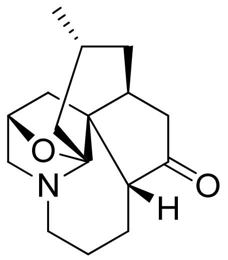

2

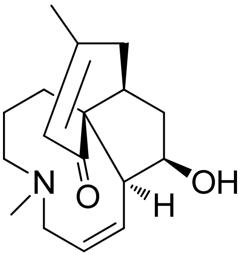

3

Structures of compounds **1**–**3**

*To whom correspondence should be addressed. E-mail: qinshizhao@mail.kib.ac.cn

Contents of Supplementary Material

No.	Contents	page
Figure S1	^{1}H NMR spectrum of compound **1**	3
Figure S2	^{13}C NMR spectrum of compound **1**	3
Figure S3	HSQC spectrum of compound **1**	4
Figure S4	^{1}H–^{1}H COSY spectrum of compound **1**	4
Figure S5	HMBC spectrum of compound **1**	5
Figure S6	ROESY spectrum of compound **1**	5
Figure S7	HRESIMS spectrum of compound **1**	6
Figure S8	^{1}H NMR spectrum of compound **2**	6
Figure S9	^{13}C NMR spectrum of compound **2**	7
Figure S10	HSQC spectrum of compound **2**	7
Figure S11	^{1}H–^{1}H COSY spectrum of compound **2**	8
Figure S12	HMBC spectrum of compound **2**	8
Figure S13	ROESY spectrum of compound **2**	9
Figure S14	HRESIMS spectrum of compound **2**	9
Figure S15	^{1}H NMR spectrum of compound **3**	10
Figure S16	^{13}C NMR spectrum of compound **3**	10
Figure S17	HSQC spectrum of compound **3**	11
Figure S18	^{1}H–^{1}H COSY spectrum of compound **3**	11
Figure S19	HMBC spectrum of compound **3**	12
Figure S20	ROESY spectrum of compound **2**	12
Figure S21	HRESIMS spectrum of compound **3**	13

Figure S1

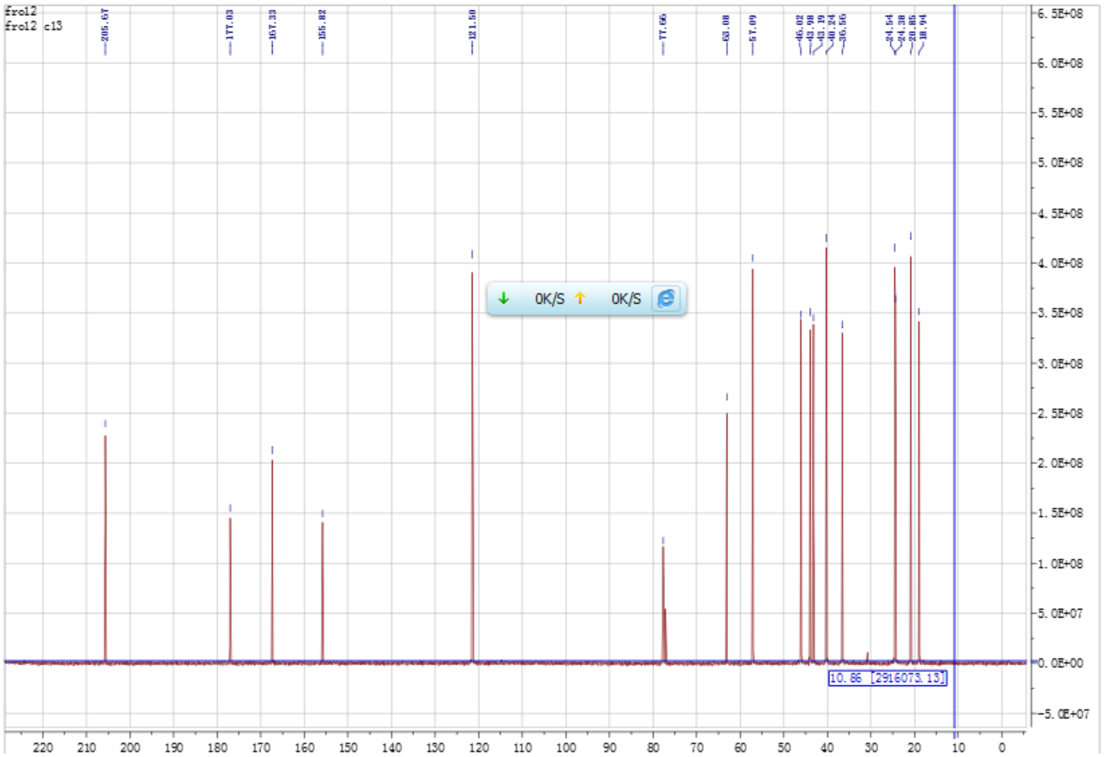

Figure S2

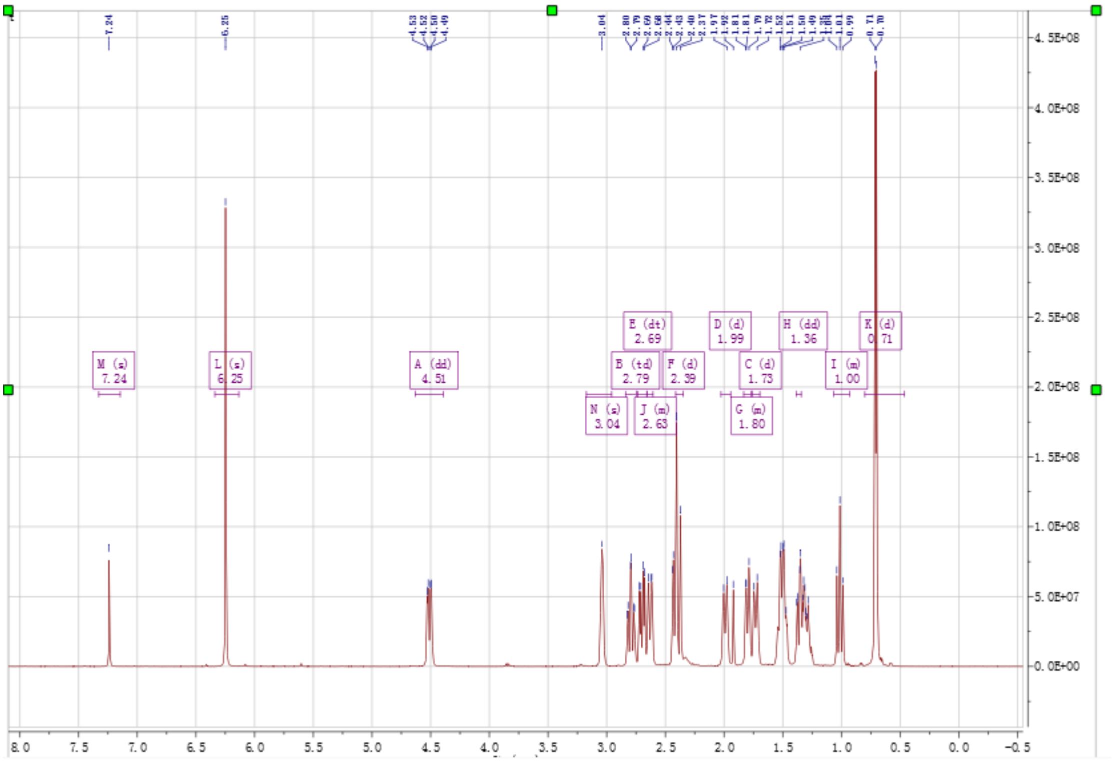

Figure S3

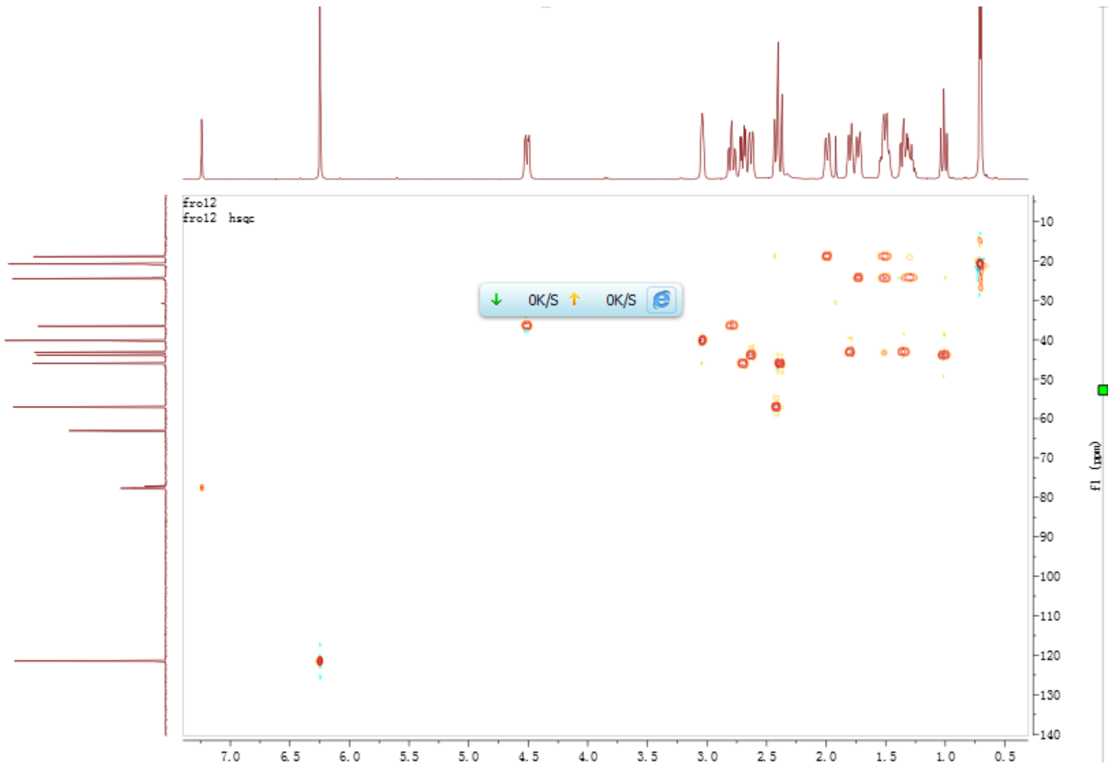

Figure S4

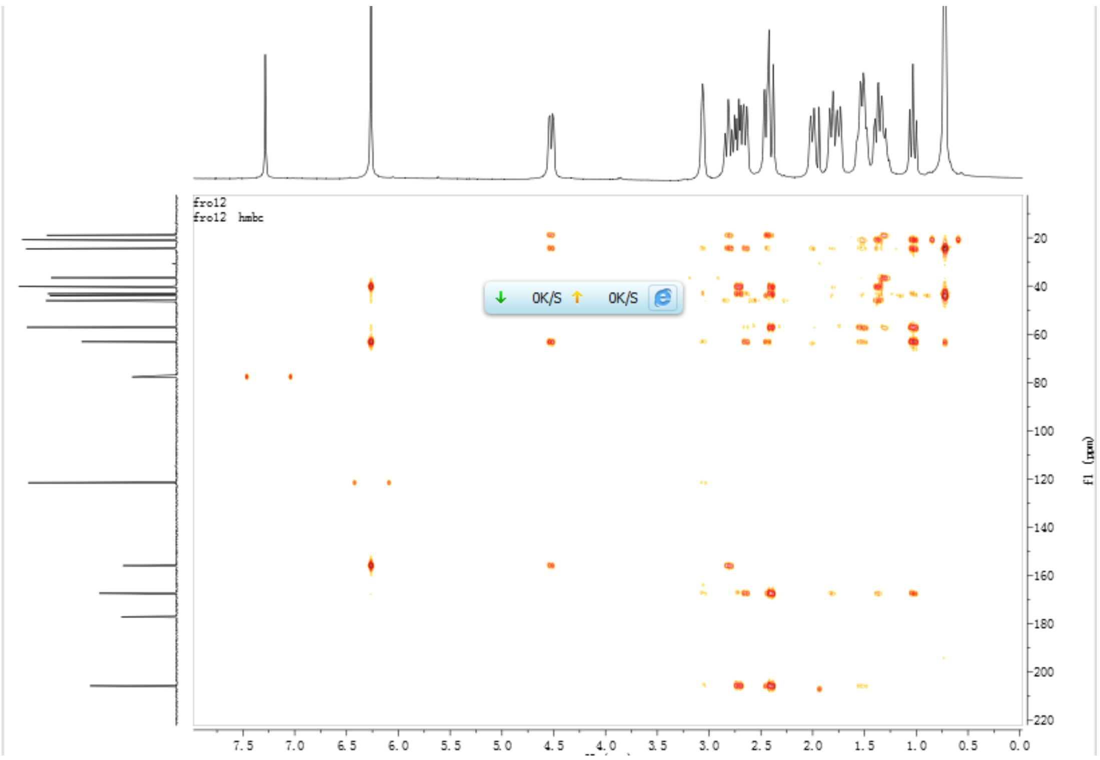

Figure S5

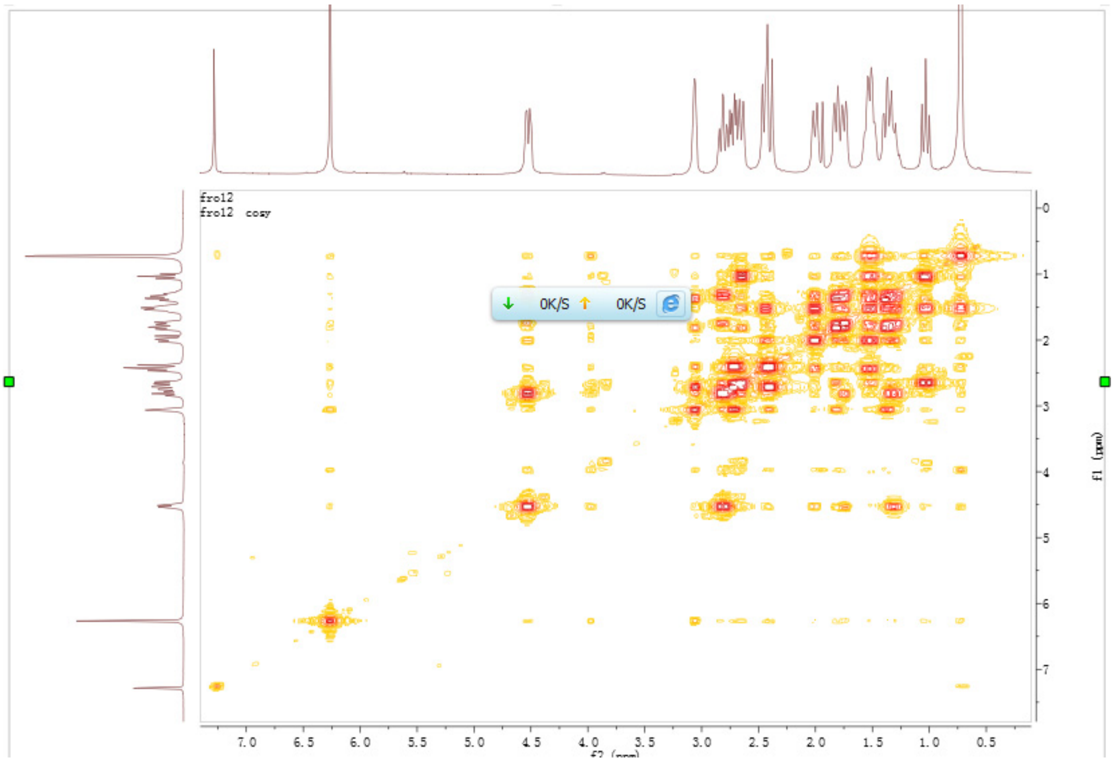

Figure S6

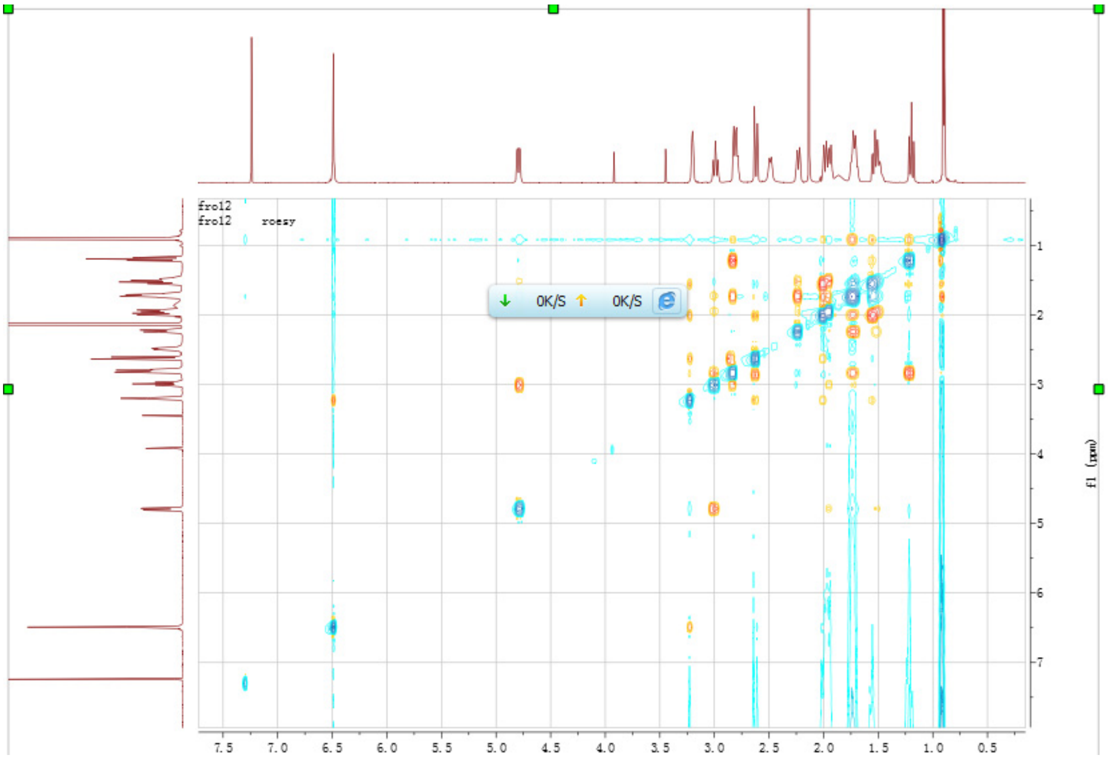

Figure S7

Elemental Composition Report

Single Mass Analysis (displaying only valid results)
Tolerance = 10.0 PPM / DBE: min = 0.5, max = 40.0
Selected filters: None

Monoisotopic Mass, Odd and Even Electron Ions
15 formula(e) evaluated with 1 results within limits (up to 51 closest results for each mass)
Elements Used:
C: 0-200 H: 0-400 N: 1-1 O: 0-3

fro-12
11:45:12 07-Feb-2012
Voltage EI+

KIB
M120207EA-02AFAMM 16 (1.469)

Autospec Premier
P776
197

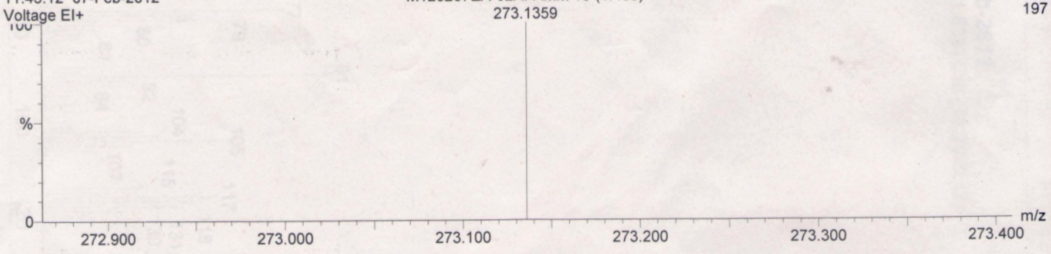

Minimum:				0.5		
Maximum:		100.0	10.0	40.0		
Mass	Calc. Mass	mDa	PPM	DBE	i-FIT	Formula
273.1359	273.1365	-0.6	-2.2	8.0	5546106.0	C16 H19 N O3

Figure S8

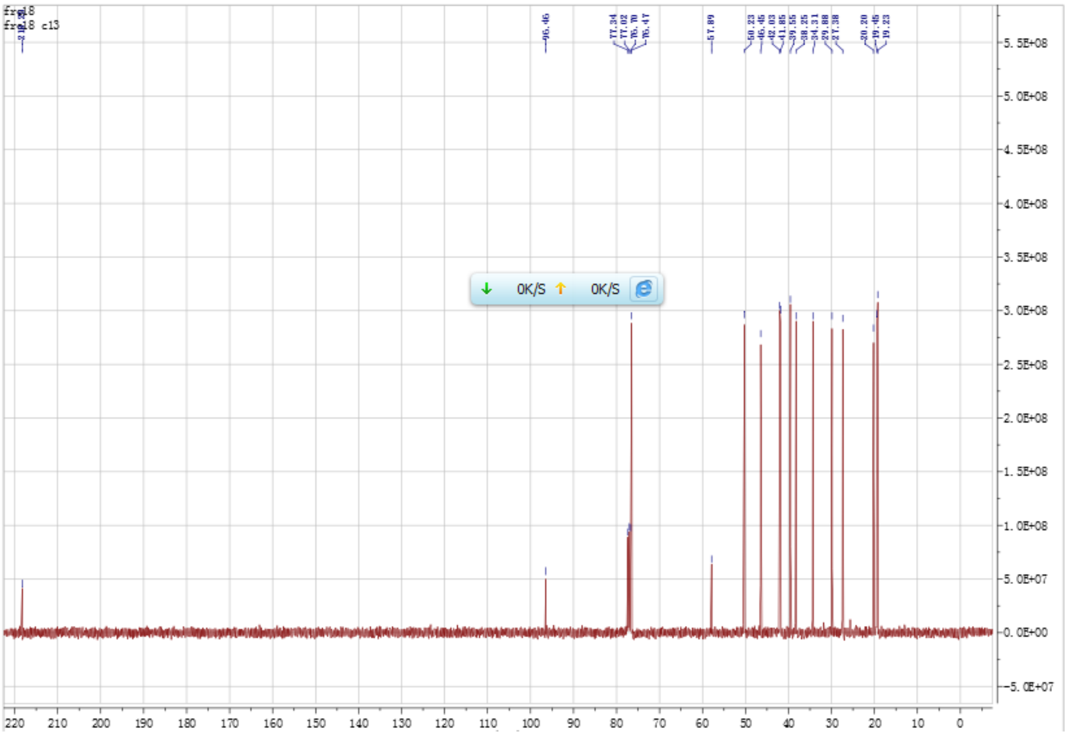

Figure S9

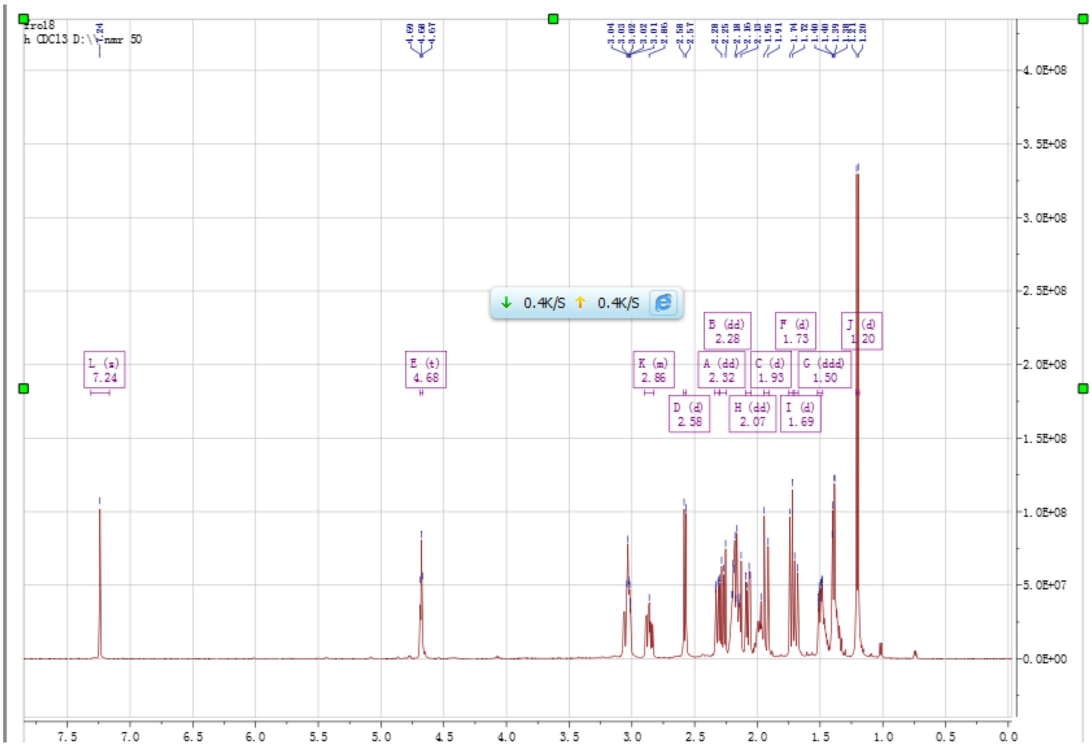

Figure S10

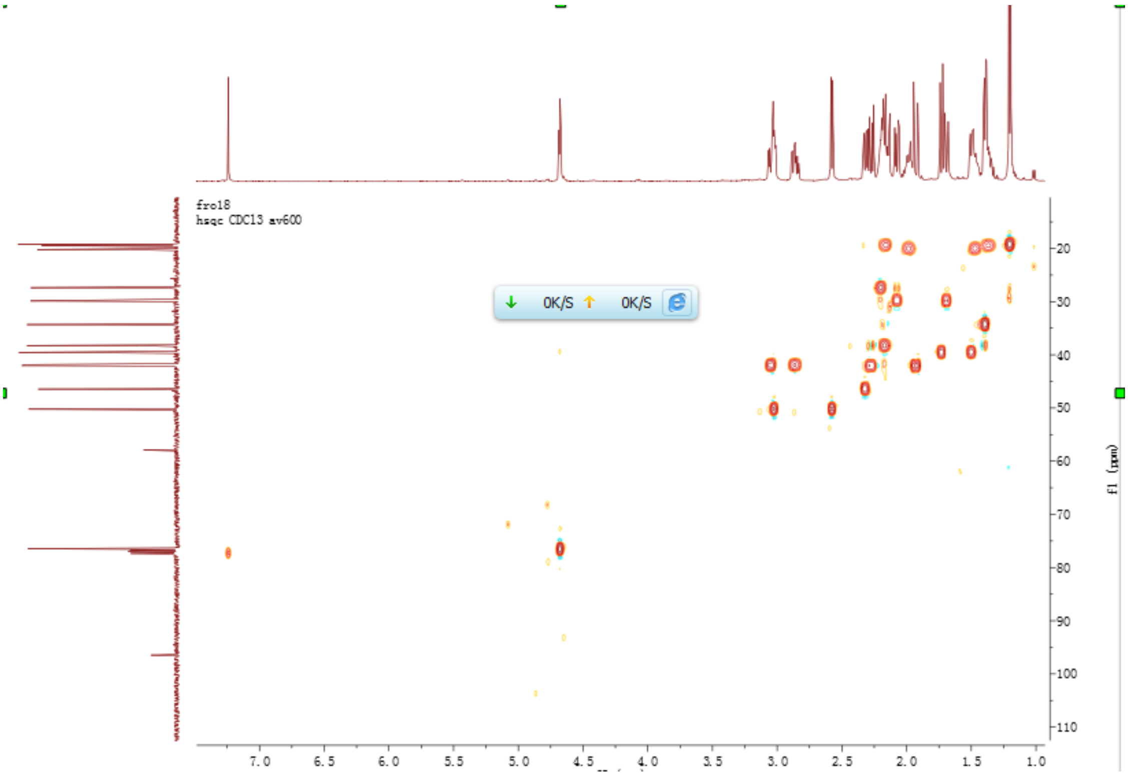

Figure S11

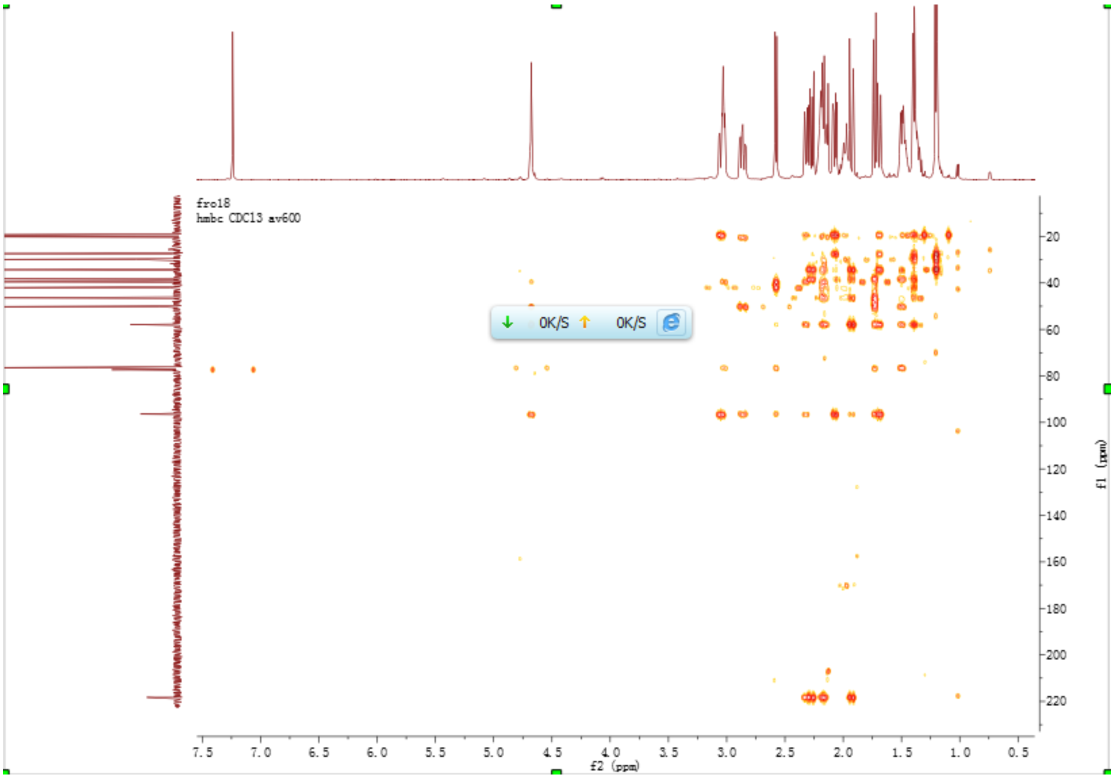

Figure S12

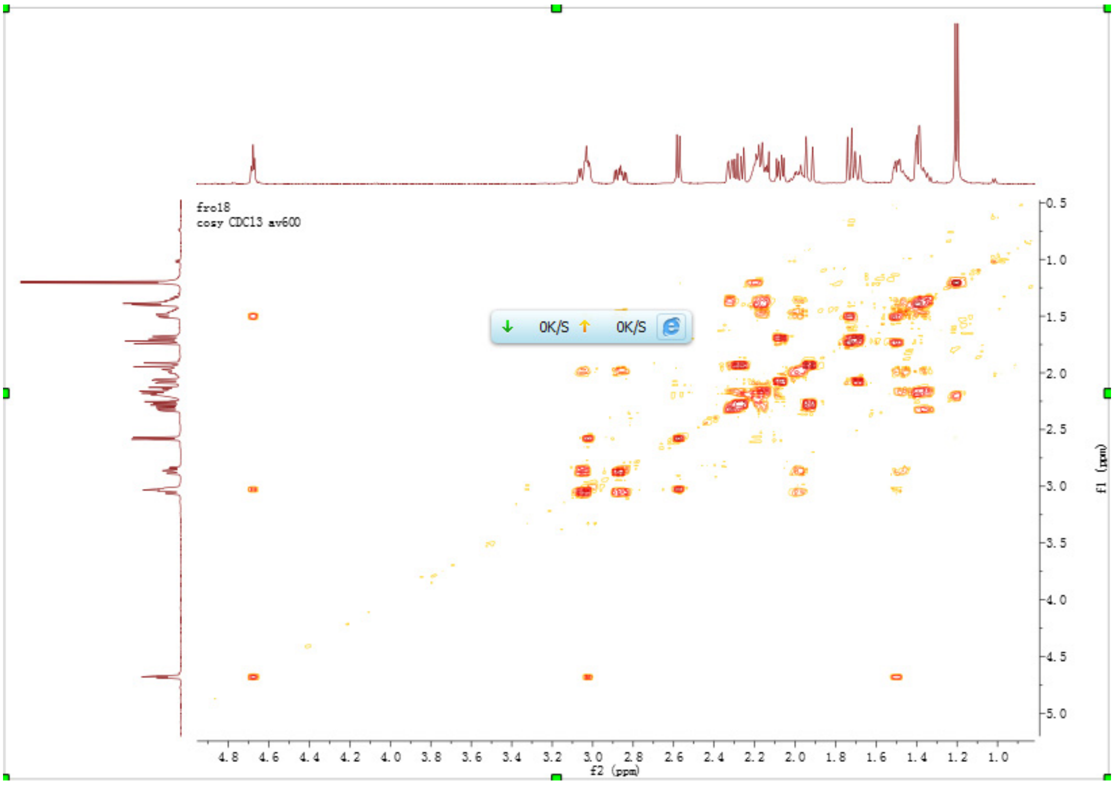

Figure S13

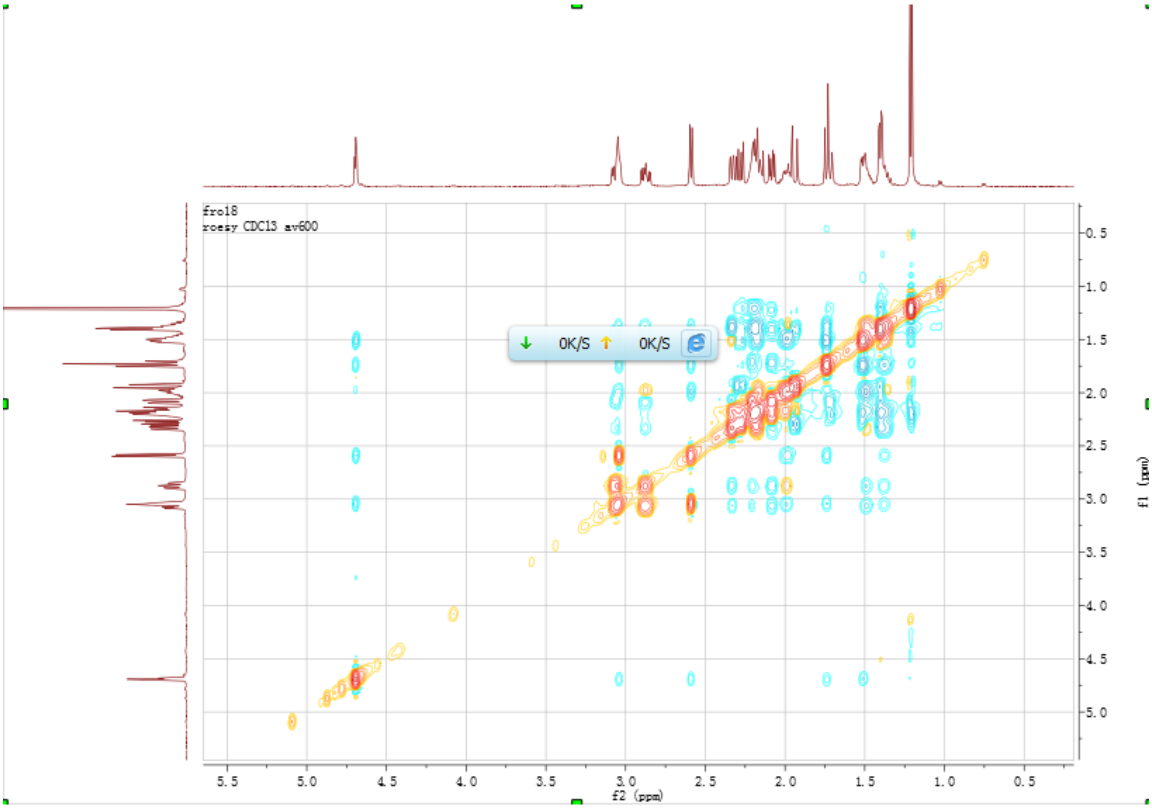

Figure S14

Elemental Composition Report

张雪媛-HRZ21 64-

Page 1

Single Mass Analysis
Tolerance = 10.0 mDa / DBE: min = 0.5, max = 120.0
Selected filters: None

Monoisotopic Mass, Odd and Even Electron Ions
15 formula(e) evaluated with 1 results within limits (up to 51 closest results for each mass)
Elements Used:
C: 0-200 H: 0-400 N: 1-1 O: 0-3

fro-18
11:13:29 03-May-2012
Voltage EI+

KIB
M120504EA-16AFAMM 14 (1.286)

Autospec Premier
P776
7.02e3

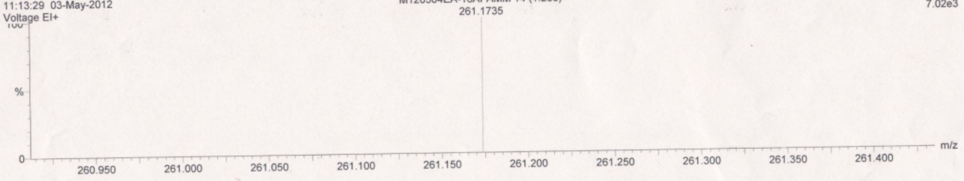

Minimum:				0.5		
Maximum:		10.0	10.0	120.0		
Mass	Calc. Mass	mDa	PPM	DBE	i-FIT	Formula
261.1735	261.1729	0.6	2.3	6.0	5549517.5	C16 H23 N O2

Figure S15

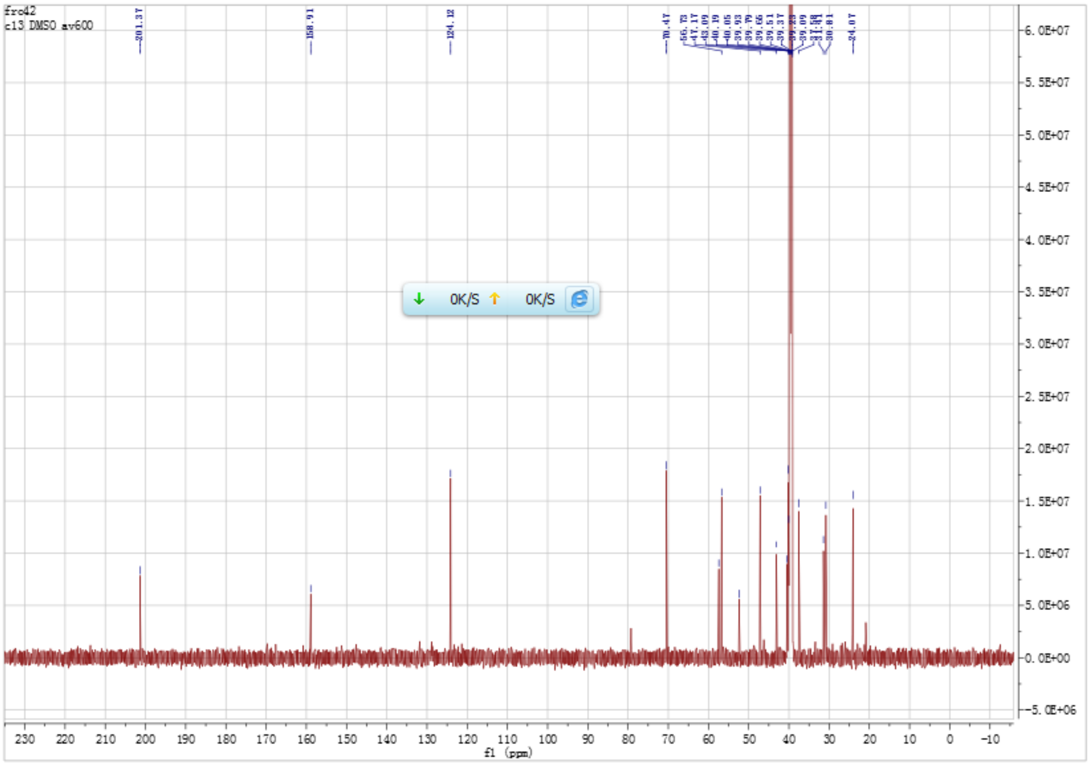

Figure S16

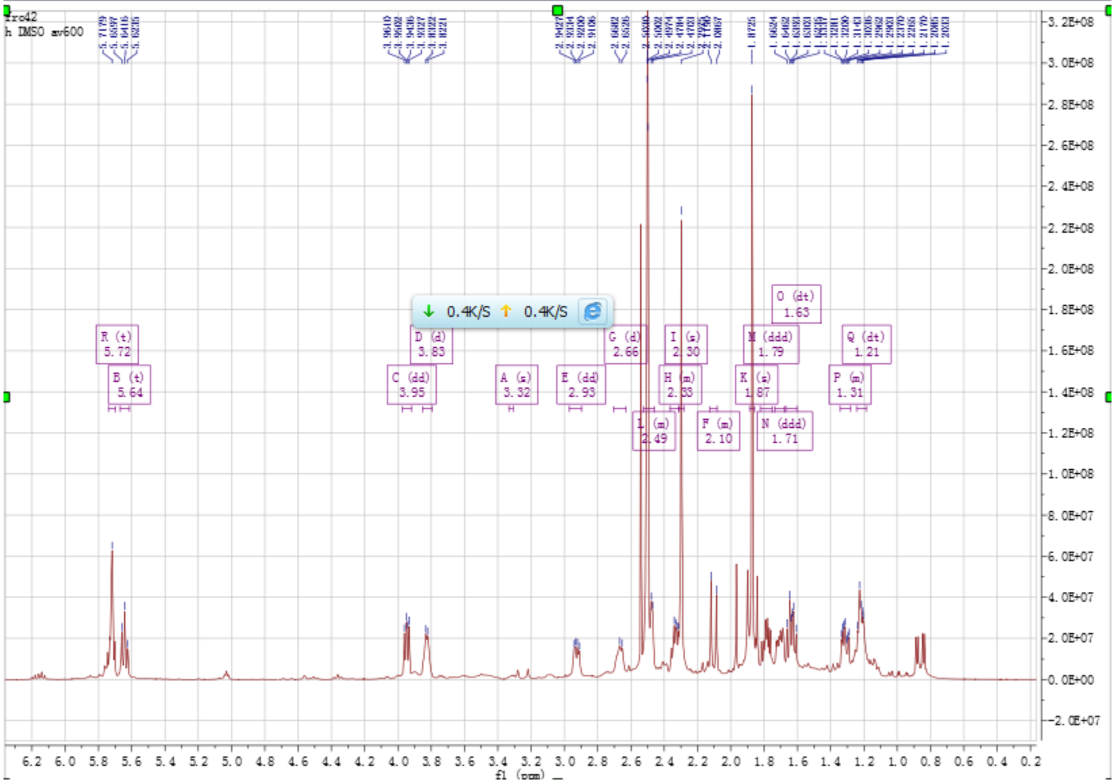

Figure S17

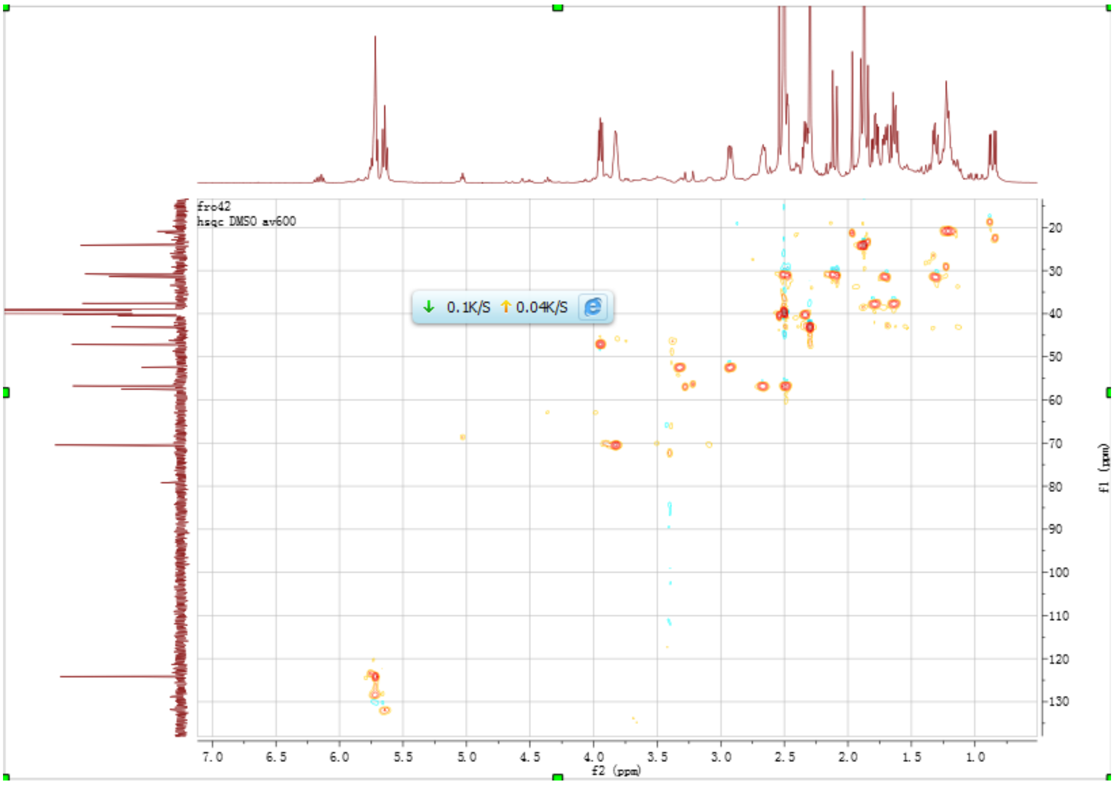

Figure S18

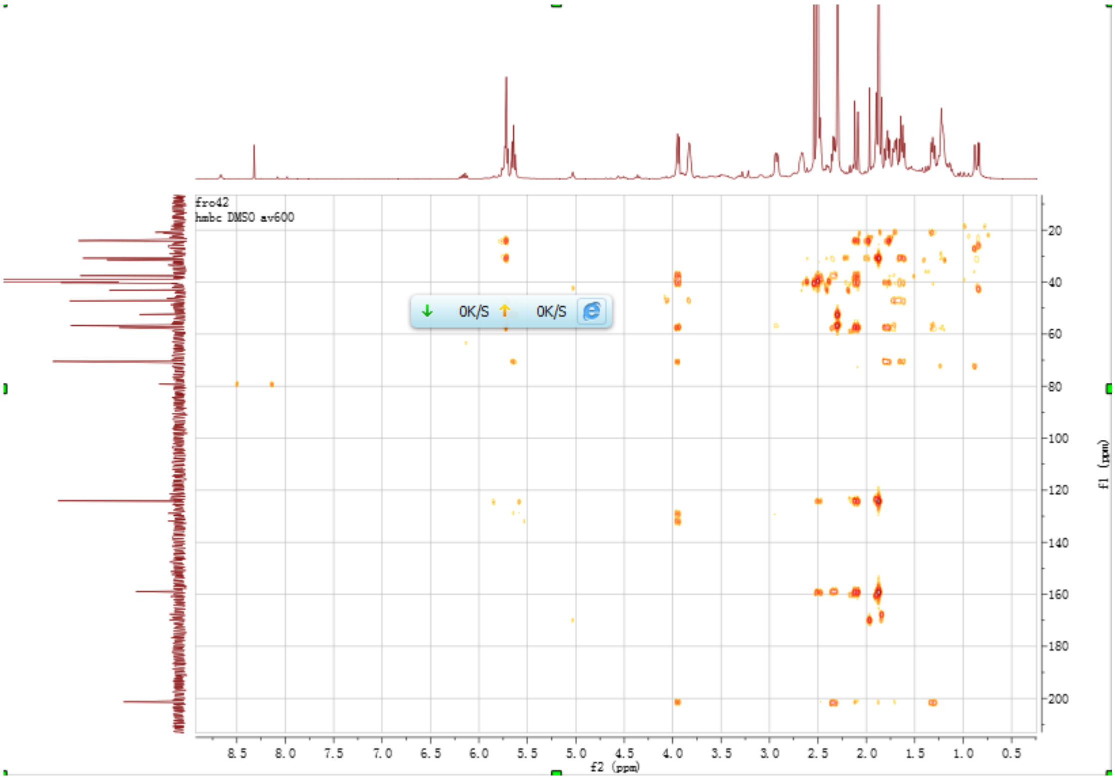

Figure S19

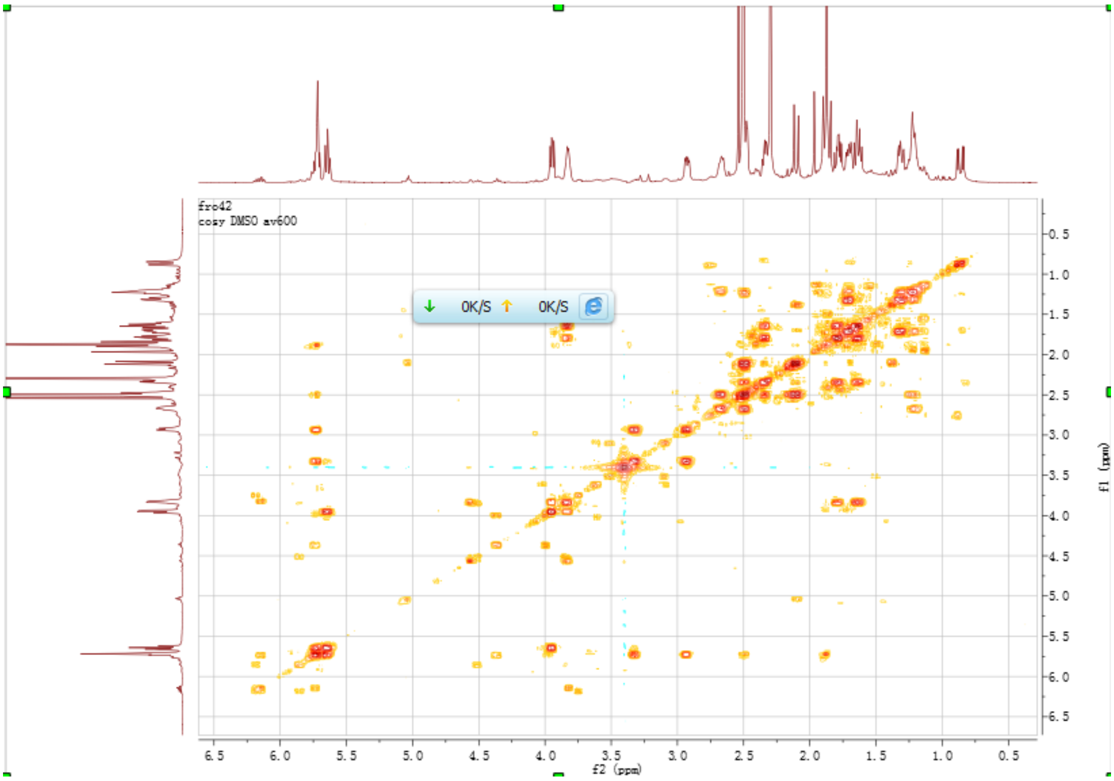

Figure S20

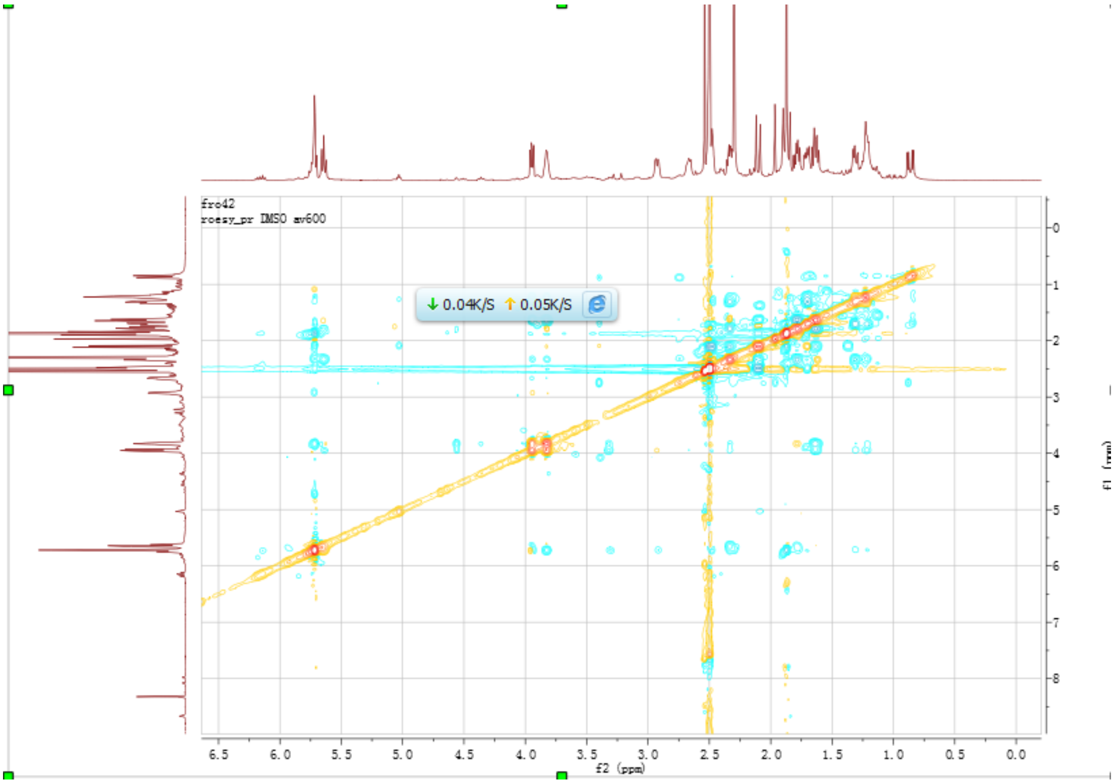

Figure S21

Elemental Composition Report

Page 1

Single Mass Analysis
Tolerance = 10.0 PPM / DBE: min = -10.0, max = 120.0
Selected filters: None

Monoisotopic Mass, Odd and Even Electron Ions
60 formula(e) evaluated with 1 results within limits (up to 51 closest results for each mass)
Elements Used:
C: 0-200 H: 0-400 N: 0-2 O: 0-3

fro-42
09:50:20 13-Nov-2012
Voltage EI+

KIB
M121113EA-02AFAMM 11 (1.010)

Autospec Premier
P776
15.7

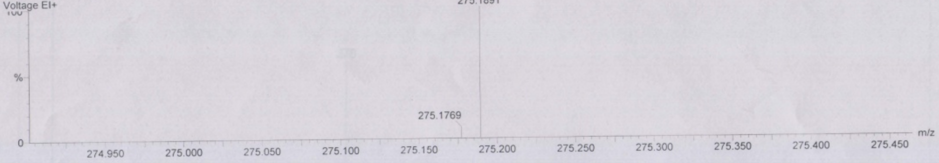

Minimum:				-10.0		
Maximum:		100.0	10.0	120.0		
Mass	Calc. Mass	mDa	PPM	DBE	i-FIT	Formula
275.1891	275.1885	0.6	2.2	6.0	5546026.5	C17 H25 N O2
